# Retention as an integrated biodiversity conservation approach for continuous-cover forestry in Europe

**DOI:** 10.1007/s13280-019-01190-1

**Published:** 2019-05-04

**Authors:** Lena Gustafsson, Jürgen Bauhus, Thomas Asbeck, Andrey Lessa Derci Augustynczik, Marco Basile, Julian Frey, Fabian Gutzat, Marc Hanewinkel, Jan Helbach, Marlotte Jonker, Anna Knuff, Christian Messier, Johannes Penner, Patrick Pyttel, Albert Reif, Felix Storch, Nathalie Winiger, Georg Winkel, Rasoul Yousefpour, Ilse Storch

**Affiliations:** 1 Department of Ecology, P.O Box 7044, 750 07 Uppsala, Sweden; 2 Chair of Silviculture, Faculty of Environment and Natural Resources, University of Freiburg, Tennenbacherstr. 4, 79106 Freiburg, Germany; 3 Chair of Forestry Economics and Forest Planning, Faculty of Environment and Natural Resources, University of Freiburg, Tennenbacherstr. 4, 79106 Freiburg, Germany; 4 Chair of Wildlife Ecology and Management, Faculty of Environment and Natural Resources, University of Freiburg, Tennenbacherstr. 4, 79106 Freiburg, Germany; 5 Chair of Remote Sensing and Landscape Information Systems, Faculty of Environment and Natural Resources, University of Freiburg, Tennenbacherstr. 4, 79106 Freiburg, Germany; 6 Department of Biometry and Environmental System Analysis, Faculty of Environment and Natural Resources, University of Freiburg, Tennenbacherstr. 4, 79106 Freiburg, Germany; 7 Chair of Geobotany, Faculty of Biology, University of Freiburg, Schänzlestr. 1, 79104 Freiburg, Germany; 8 Abteilung Waldnaturschutz, Baden-Württemberg Forest Research Institute (FVA), Wonnhaldestraße 4, 79100 Freiburg, Germany; 9 Chair of Nature Conservation and Landscape Ecology, Faculty of Environment and Natural Resources, University of Freiburg, Tennenbacherstr. 4, 79106 Freiburg, Germany; 10 Centre for Forest Research, Université du Québec à Montréal, PO Box 8888, Centre-Ville Station, Montreal, QC H3C 3P8 Canada; 11 Institut des sciences de la forêt tempérée (ISFORT), Université du Québec en Outaouais, 58 Rue Principale, Ripon, QC J0V 1V0 Canada; 12 Chair of Site Classification and Vegetation Science, Faculty of Environment and Natural Resources, University of Freiburg, Tennenbacherstr. 4, 79106 Freiburg, Germany; 13 Resilience Programme, European Forest Institute, Platz der Vereinten Nationen 7, 53133 Bonn, Germany

**Keywords:** Biodiversity, Habitat tree, Retention forestry, Temperate forests, Uneven-aged management

## Abstract

Retention forestry implies that biological legacies like dead and living trees are deliberately selected and retained beyond harvesting cycles to benefit biodiversity and ecosystem functioning. This model has been applied for several decades in even-aged, clearcutting (CC) systems but less so in uneven-aged, continuous-cover forestry (CCF). We provide an overview of retention in CCF in temperate regions of Europe, currently largely focused on habitat trees and dead wood. The relevance of current meta-analyses and many other studies on retention in CC is limited since they emphasize larger patches in open surroundings. Therefore, we reflect here on the ecological foundations and socio-economic frameworks of retention approaches in CCF, and highlight several areas with development potential for the future. Conclusions from this perspective paper, based on both research and current practice on several continents, although highlighting Europe, are also relevant to other temperate regions of the world using continuous-cover forest management approaches.

**Electronic supplementary material:**

The online version of this article (10.1007/s13280-019-01190-1) contains supplementary material, which is available to authorized users.

## Introduction

Forests are the dominant vegetation form globally, and play a key role in conserving biodiversity as well as safeguarding ecosystem services (Millennium Ecosystem Assessment 2005). Strictly protected areas provide high conservation value, but are insufficient for preserving biodiversity due to their limited area, poor connectivity and high rates of anthropogenic disturbance worldwide (Ellis et al. 2013). In the multiple-use landscapes of Europe, the integration of conservation into forest planning and management is crucial to better achieve desired biodiversity goals, like those set forth in the EU Forest and Biodiversity strategies (European Commission 2013, 2015).

**Fig. 1:**
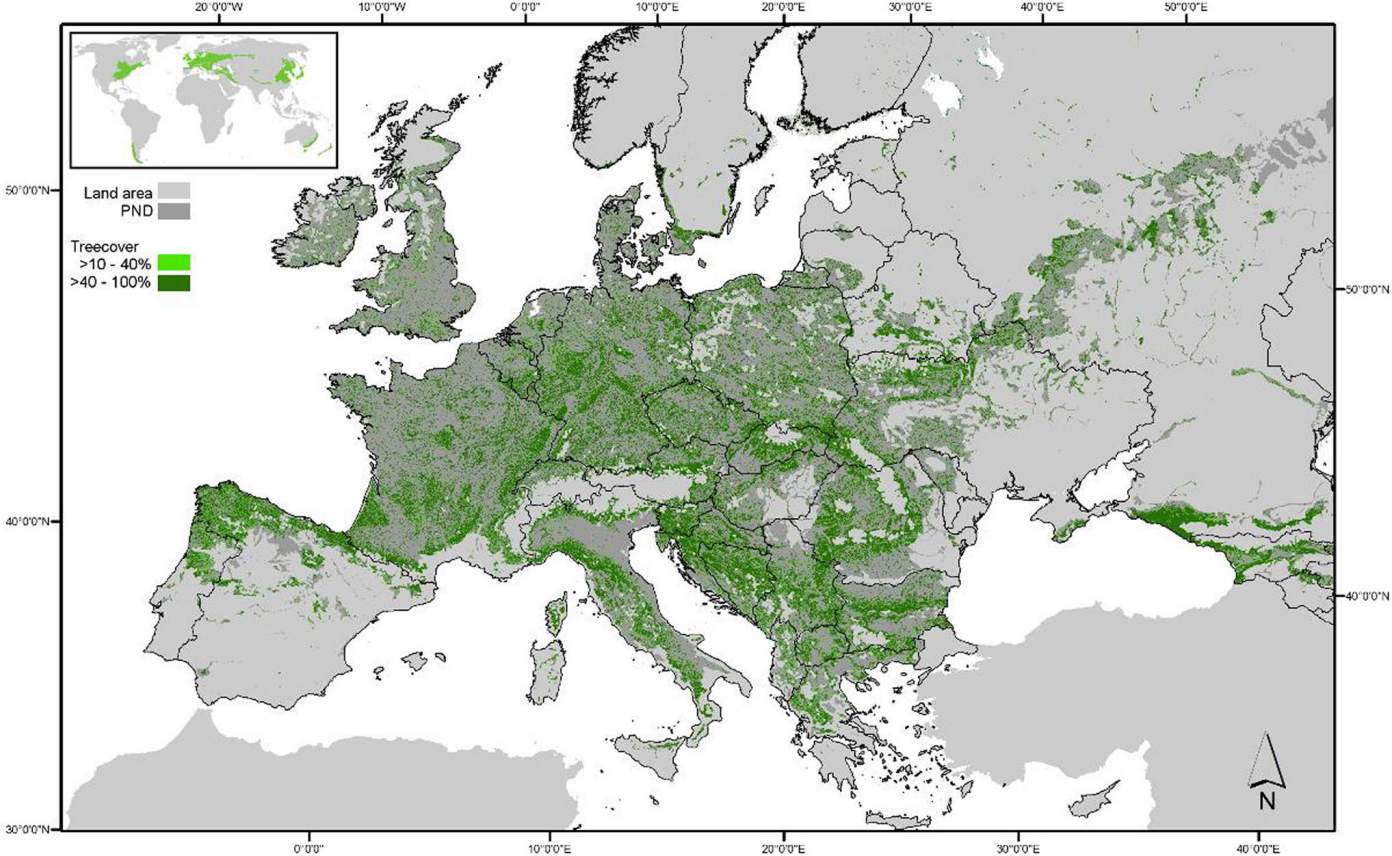
Distribution of temperate deciduous and mixed forests in Europe (inlet: global distribution). The current temperate forest distribution in Europe is shown in green (light green 10–40%, dark green > 40% tree cover), with the potential natural distribution (PND) shown in dark grey. Information presented in the inset is based on Terpsichores 2017; Wikimedia Commons: https://commons.wikimedia.org/wiki/File:Biome_map_04.svg). See Appendix S5 for maps of Europe with higher resolution, finer classification and data sources

Temperate forests are common throughout Central and Eastern Europe, stretching into the Balkans, Italy, Spain, the Caucasus and west Russia (Fig. 1), with beech (*Fagus sylvatica*) as a main natural tree species (Bohn et al. 2003). Temperate forests form a large vegetation belt in the northern hemisphere but also occur in parts of South America, Australia and New Zealand.

Human pressures on forests have been more profound and long-lasting in Central Europe than in many other parts of the world, with agricultural expansion causing extensive deforestation over the centuries. Since the middle of the nineteenth century, the forest area has recovered considerably through abandonment of agricultural land and afforestation. The latter was largely carried out with fast growing conifers such as Norway spruce *Picea abies* and Scots pine *Pinus sylvestris* (McGrath et al. 2015). Currently, less than 2% of European forestland is strictly protected (Forest Europe 2015), and the major conservation approach of the EU, Natura 2000, includes areas subjected to conventional forest management (Winkel et al. 2015). Furthermore, the forest-ownership structure is multifaceted, with half of the forests privately owned and the majority of owners holding lots smaller than one hectare, resulting in a huge variation in management goals and practices (Schmitt et al. 2009). Currently in Europe, the differences between uneven-aged and even-aged management largely reflects differences between forest biomes, with clearcutting (CC) being the typical form of harvesting in the boreal regions with conifer-dominated forests (Kuuluvainen 2009), and continuous-cover forestry (CCF) being more associated with temperate, broadleaved forests (Bauhus et al. 2013). However, since the application of management practices depends on landowner objectives, there is no strict regional division between these two approaches and, as a result, transitional forms of harvesting such as shelterwood systems with short regeneration phases exist (Bauhus and Pyttel 2015).

The foundations of CCF were developed in Central Europe at the end of the nineteenth and beginning of the twentieth century (e.g. Biolley 1901; Möller 1922). It is currently a dominant forest management model in Germany, France, Switzerland and Slovenia, and is practised to some degree in other European countries. The principle attribute of CCF is the selective harvesting of individual trees or groups of trees to maintain continuous forest conditions. Recently, in several parts of Central Europe, a major goal of CCF has been the conversion of mostly even-aged conifer forests towards uneven-aged, mixed species stands of broadleaved trees (e.g. Bürgi and Schuler 2003). The deliberate retention of habitat trees (i.e. trees that provide biodiversity values; see Table 1) and dead wood, however, is a relatively recent complementary management goal (Bauhus et al. 2013).

**Table 1:** Terminology related to retention approaches in continuous-cover forestry

Terms	Definitions
Clearcutting	The harvesting of all trees at the same time
Continuous-cover forestry (CCF)	A forest management approach without clearfelling that maintains various tree ages within a stand by periodically selecting and harvesting individual trees or groups of trees. Synonym: uneven-aged management
Even-aged management	A management approach that regenerates forests through clearcutting, seed-tree systems, or short shelterwood phases resulting in stands composed of trees of a similar age (even-aged stands)
Habitat tree	A tree with special characteristics (unusual tree species, old age, microhabitats) or with good potential for developing important microhabitats, which makes it especially valuable to current or future biodiversity. Habitat trees are a main structure retained at harvest to promote biodiversity. Synonym: veteran tree
Legacy	A biological structure that persists over the harvesting phase, often a living or dead tree. It represents ecological continuity that is important to species and/or ecosystem functioning
Life-boating	The ability of trees retained at harvest to ensure survival of species from the pre-harvest phase over the regeneration phase
Tree-related microhabitat (TreM)	A structure on a living or standing dead tree that is particularly important to a species as a food source, shelter or other habitat requirements. Examples include cavities, burrs and cankers
Retention forestry	A forest management approach based on the long-term retention of structures and organisms, such as living and/or dead trees as well as small areas of intact forest, at the time of harvest. This approach aims to achieve a level of continuity in forest structure, composition and complexity that promotes biodiversity and sustains ecological function at different spatial scales. See Appendix S1 for more detailed terminology
Uneven-aged management	See continuous-cover forestry
Veteran tree	See habitat tree

Retention forestry aims at integrating key biodiversity structures into production forests, and is currently used mostly in CC in North America, Europe, and parts of Australia and South America (Gustafsson et al. 2012). It implies the long-term retention of structures and organisms, such as live and dead trees and small areas of intact forest, at the time of harvest with the aim to benefit biodiversity and ecosystem functioning (Gustafsson et al. 2012). This practice (also known as variable retention or green-tree retention; Table 1 and Appendix S1) was introduced in the Pacific Northwest of North America about 30 years ago not only in response to the observed negative ecological impacts of CC, but also due to increased awareness about the structural diversity associated with disturbances (Franklin 1989). Biodiversity-related legislation, its interpretation by courts and the demand for products from sustainably managed forests have been strong drivers in the implementation of retention forestry (e.g. Cashore et al. 2004).

An overview and discussion of retention approaches in CCF is timely and much needed since the practical application in this forest management system is increasing. A logical starting point is an evaluation of the relevance of the knowledge base compiled for retention in CC, including several overview and review papers (e.g. Rosenvald and Lõhmus 2008; Gustafsson et al. 2012; Lindenmayer et al. 2012; Fedrowitz et al. 2014; Mori and Kitagawa 2014). We focus our overview on Europe since implementation of CCF in this region is widespread, and retention approaches have been introduced into forest management in several countries, especially in Central Europe (Kraus and Krumm 2013). The interest in CCF including retention is also increasing in the boreal region of Europe (Peura et al. 2018). In this perspective paper, we build on our experience and insights from research within the field, and our general understanding of forest and conservation policy and practice from different countries, mainly in Europe but also on other continents. Based on this, we reflect on the ecological foundations and socio-economic frameworks of retention approaches in CCF, and highlight several areas with development potential for the future. In particular we point to specific circumstances for the temperate forests of Europe but since ecological patterns and processes, and harvesting systems show many similarities over temperate forests on different continents (e.g. Mitchell et al. 2006), our conclusions are also relevant to other parts of the world. Contrary to earlier overviews and systematic reviews on retention forestry, we emphasize its application in one type of forest management system (CCF), and we critically discuss the relevance of the current evidence-based knowledge of retention forestry for this system, and reflect on the influence of specific socio-economic contexts in Europe for the application of retention forestry in CCF.

## Focus on habitat trees and dead wood

**Fig. 2:**
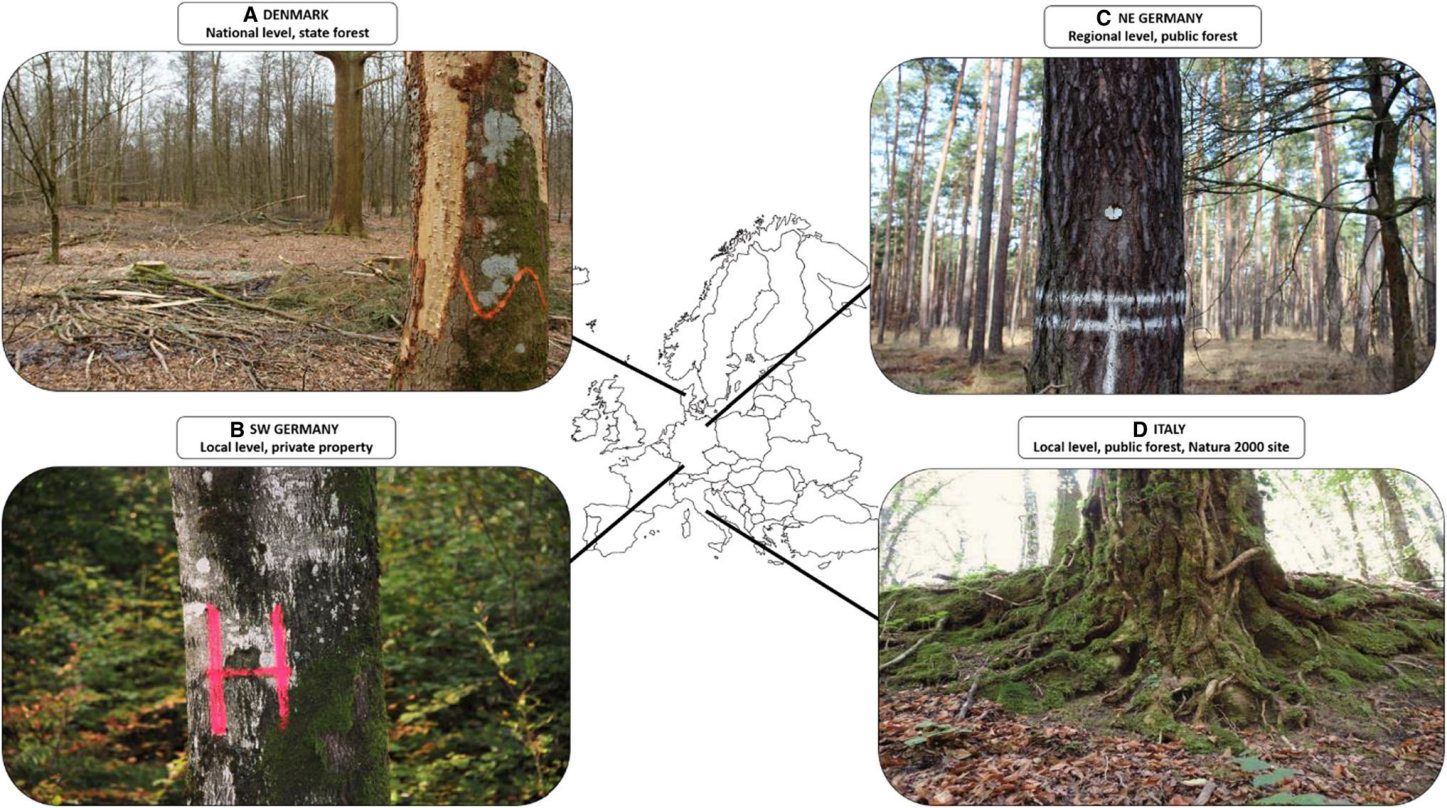
Examples of retention approaches used in continuous-cover forestry in different parts of Europe, representing different scale levels and forest-owner categories. **a** Denmark. National level, state forest. Retention actions are mandatory for harvest operations across the ca. 110 000 ha of forests belonging to the Danish state, which are managed through close-to-nature forestry. Examples include the retention of at least five habitat trees per ha and the intentional injury of three trees per ha—in some stands—to speed up the decay process. Photo: Lena Gustafsson. **b** SW Germany. Local level, private property (230 ha). Habitat trees, especially those with cavities, are marked and excluded from harvesting. An energy company has reimbursed the forest owner for saving a retention patch as compensation for a wind-park in the vicinity. Photo: Hermann Rodenkirchen. **c** NE Germany. Regional level, public forest (430 000 ha). During the “Methusalem project”, more than 200 000 trees have been retained in the Public Federal State of Brandenburg, for conservation and aesthetic reasons. Photo: Gernod Bilke. **d** Italy. Local level, public forest, Natura 2000 site (3100 ha). Retention approaches were introduced in the Molise Region, central Italy, about 10 years ago as a way of sustainable forest management. Actions include retaining habitat trees, coarse standing dead trees and fallen dead wood, and all cavity trees. Photo: Marco Basile. For more information, see Appendix S6

Retention in CCF of temperate European forests is largely focused on habitat trees and dead wood (Kraus and Krumm 2013), as manifested in the national certifications standards (FSC/PEFC) for European countries in which CCF is practised (e.g. Austria, Denmark, France, Germany, Switzerland). These standards show that common retention actions are to leave large living trees (habitat trees, veteran trees; Table 1), and both standing dead trees and fallen dead wood on site (Appendices S2, S3). Leaving larger forest patches, as is the prevailing practice in CC (e.g. Gustafsson et al. 2012), is presently less common. From a practical perspective, the high emphasis in CCF on individual retention elements involves planning that is often highly detailed, and includes the careful selection of individual trees, and small groups of trees based on a range of attributes such as microhabitats (e.g. Larrieu et al. 2018). However, the implementation in temperate forests of Europe varies among regions and forest-ownership categories (see examples in Fig. 2), and regarding retention levels, indicated by the national certification standards, where the density of living habitat trees varies between 1 and 10 ha^−1^, and dead wood varies between 1 and 20 trees ha^−1^ (Appendices S2, S3). Comparison of retention levels among countries should be made with caution since information is lacking on forest management systems and actual practices in different countries. Thus, retention prescriptions specific to CCF cannot be assessed.

## What can be learned from retention in clearcutting forestry?

The peer-reviewed literature on retention harvesting from around the world is extensive (Lindenmayer et al. 2012), and several large-scale retention experiments have been established (Gustafsson et al. 2012). Some meta-analyses have also been conducted including data from several biomes and continents (Rosenvald and Lõhmus 2008; Fedrowitz et al. 2014; Mori and Kitagawa 2014; Basile et al. 2019). However, the information gained from these meta-analyses for retention in CCF is limited since they do not specifically target biodiversity associated with habitat trees and dead wood, the current core of CCF, but instead focus on the flora and fauna of retained forest patches, often in comparison to clearcuts without such patches. For instance, Fedrowitz et al. (2014) in the largest meta-analysis to date with > 900 comparisons between patches retained at harvest and clearcuts found a higher species richness in patches. A positive response regarding species richness was also found in comparisons between retained patches and mature forests (Fedrowitz et al. 2014; Mori and Kitagawa 2014). Although results from these meta-analyses are highly valuable, they provide little guidance for retention in CCF.

**Fig. 3:**
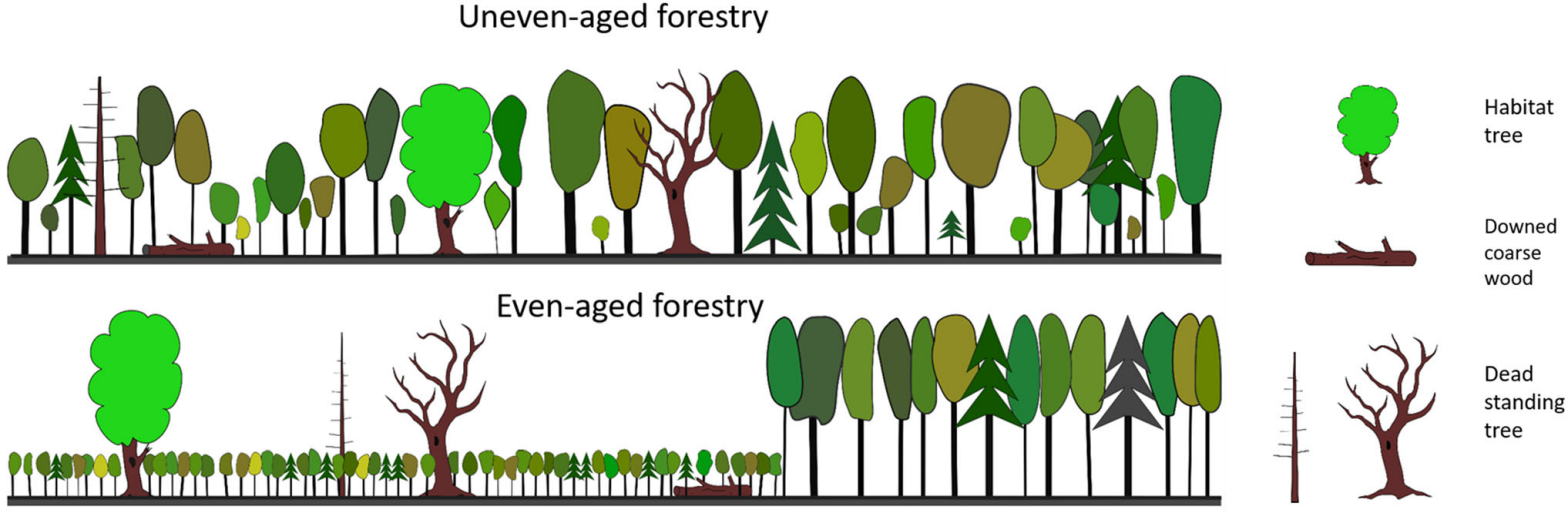
Retained structural elements (habitat trees, standing and fallen dead wood) in uneven-aged, continuous-cover forestry (upper panel) and even-aged clearcutting forestry (lower panel)

The role of microclimate, in particular light, has been recognized to only a limited extent as a key aspect for biodiversity in CCF (but see Mölder et al. 2019). Over time, canopy cover in CCF is considerably more dense and homogenous, vertically and horizontally compared to CC where early open phases following harvest are successively replaced with higher and more closed forest (Table 2; Fig. 3). Nevertheless, light availability within stands managed through selective harvesting approaches may also vary greatly at small spatial scales (e.g. Brunet et al. 2010), and affect biodiversity. For instance, current light conditions surrounding habitat trees may can be important for the diversity of saproxylic beetles (Koch Widerberg et al. 2012), and such species may also be affected by past light conditions (Miklín et al. 2018). Light can also be an important factor for biodiversity associated with dead wood (Horak et al. 2014; Seibold et al. 2016). Since retained habitat trees in CCF are, at least initially, less exposed to wind and direct sunlight when compared to CC, they may also be more protected and less susceptible to mortality (Carter et al. 2017). At the landscape scale, CCF practised over large areas may lead to a relatively homogenous and continuous tree cover with comparatively small contrasts between stands (Schall et al. 2018). Unlike the CC where retained elements often are in distinct patches, the habitat trees and dead wood in CCF are embedded, often dispersed, in a “non-hostile” forest matrix with little microclimatic contrast (Table 2; Fig. 3). Consequently, a knowledge base different from that currently used in CC retention forestry will be needed for CCF retention.

**Table 2:** Continuous-cover forestry and clearcutting compared regarding environmental conditions, type of retention and the evidence base for retention actions. See also Fig. 3

	Continuous-cover (uneven-aged) forestry (CCF)	Clearcutting (even-aged) forestry (CC)
Habitat suitability	Mostly suitable for forest interior species, including such that are promoted by long continuity in tree cover	Suitable for disturbance-promoted species (following harvest) and forest interior species (soon before harvest) but less so for species that are promoted by long continuity in tree cover
Retention elements	Currently in temperate Europe a large focus on habitat trees and dead wood, single trees to small groups of trees (< 0.2 ha)	Single trees to larger aggregate patches (> 1 ha) (mainly in boreal Europe, North America and Australia)
Silvicultural regeneration method	Single tree and group selection, shelterwood systems with long regeneration phases	Clearcutting, seed-tree systems
Harvesting intervals (years)	5–30	50–100
Matrix of retention elements	Retention elements embedded in non-hostile matrix with small or no microclimatic contrast	Retention elements, at least soon after harvest, embedded in hostile matrix with strong microclimatic contrast
Contrast between retention elements and surrounding	Forest interior conditions surrounding the retention elements	Open conditions outside retained patches (at least soon after harvest)
Landscape considerations for retention actions	No landscape considerations owing to small retention elements as well as (for temperate Europe) small forest properties and diverse forest-owner objectives	Retention elements sometimes planned to provide connectivity and to offer protective functions, for example as buffer strips for streams. Often large, industrial forest owners
Light conditions	Mostly shady	Variation in light intensity over the rotation period
Wind disturbance	Moderate impact on retention elements	Strong impact on retained trees and patches in recently harvested stands
Evidence base for effectiveness of retention approaches	Very few studies specifically focused on retention of habitat trees and dead wood. Systematic reviews and meta-analyses are lacking	A large amount of literature on retention actions, including several overview papers, systematic reviews and meta-analyses

## Retention elements and biodiversity

Numerous studies have demonstrated the great importance of large trees (e.g. Lindenmayer 2017; Prevedello et al. 2018) and dead wood (e.g. Roth et al. 2018) to biodiversity, but more specific insights into their role in retention systems in temperate forests are limited (but see Vítková et al. 2018; Asbeck et al. 2019). Largely independent of the silvicultural system, studies from European forests have indicated that trees serving conservation purposes can improve biodiversity by increasing habitat diversity (Müller et al. 2014; Gutzat and Dormann 2018), and that the abundance of tree-related microhabitats (e.g. structures on individual trees of importance to biodiversity; Table 1) is lower in production forests than in protected forests (Vuidot et al. 2011). In addition, single trees in closed canopy forests have been found to act as stepping stones and habitats for certain invertebrate populations (Müller and Gossner 2007). Microhabitats, such as insect galleries (Regnery et al. 2013), root buttress holes (Basile et al. 2017) and cavities (Cockle et al. 2011), although studied for a long time, have gained increasing research attention during the last years (e.g. Paillet et al. 2017; Larrieu et al. 2018). Moreover, old trees are important to many species, like bats (Regnery et al. 2013), forest-specialist birds (Ameztegui et al. 2018) and salamanders (Basile et al. 2017), while dead wood promotes saproxylic species in production forests (e.g. Seibold et al. 2015). In contrast, CCF without retention has been shown to adversely affect saproxylic beetles (Gossner et al. 2013). Overall, numerous studies in Europe on old trees, dead wood and their microhabitats, although not specifically addressing retention, form an important research base for retention practices.

## Ecological objectives and motives

Retention approaches can be integrated into all types of forests and silvicultural systems (Lindenmayer et al. 2012), and the three main objectives identified for retention forestry employing CC are valid for CCF: (1) safeguarding a continuity of structures, functions and composition, (2) increasing structural complexity, and (3) maintaining connectivity in the landscape (Franklin et al. 1997). In CCF as in most other forest management system, trees are harvested at a much younger age than their biological maturity, and thinning and other tending operations often remove tree forms and shapes of low economic value. Thus, structural diversity in the form of large old trees, dead wood, and their associated microhabitats is typically reduced (Vuidot et al. 2011). As the retained trees age, they develop old-growth structures which provide numerous microhabitats for species specialized to late-succession stages that would otherwise be lacking in production forests (Paillet et al. 2010). These legacy trees can act as “lifeboats” (Franklin et al. 2000) for old- and dead wood-dependent species, as well as help preserve a forest’s “ecological memory” (Johnstone et al. 2016). Moreover, a suitable distribution of retention elements in the landscape can improve habitat connectivity for different organisms (Müller and Gossner 2007).

## Socio-economic drivers and impacts

Regarding the temperate forests of Europe, society has placed increasingly greater importance on biodiversity and ecosystem services (e.g. recreation and carbon sequestration) than on wood production over the last decades (Borrass et al. 2017). The implementation of retention approaches in CCF has been a response to these societal expectations. Furthermore, retention has been promoted as a way to supplement strictly protected areas following conservation sector demands for an increase in the area and connectivity of protected forests (Borrass et al. 2017). These actions have resulted in relatively strong support for “integrative” forest biodiversity protection (“integrated forest management”) among foresters in several countries, for example, Germany (Maier and Winkel 2017; Blattert et al. 2018).

Research-based evidence for the importance of dead wood and habitat trees has also been a crucial factor in the adoption of retention approaches (Lindenmayer et al. 2012), as has been the European Union’s Habitats Directive, which requires Member States to participate in species protection. As for the latter, decisions by the European Court of Justice were critical to reaffirm the need to more thoroughly consider species protection in forest management, and triggered the development and implementation of retention forestry approaches to reduce legal uncertainty in forest operations in relation to impacts on protected species (Borrass et al. 2015). Retention has also been partly encouraged through forest certification, as many European national certification standards include prescriptions for maintaining habitat trees and dead wood at forest harvesting (Appendices S2, S3).

Another important driver for the implementation of retention forestry is the expected insurance value provided by forest biodiversity. In Central Europe, climate change and the associated increase in the frequency and intensity of disturbances are expected to impact forest productivity (Lindner et al. 2010). Retention forestry, by supporting forest biodiversity, could positively affect ecosystem resilience and functioning (Yachi and Loreau 1999) and thus mitigate economic losses in the future (Messier et al. 2013).

Nevertheless, retention forestry will typically lead to a loss in the net-harvestable wood volume for forest owners, since a share of the harvestable biomass (retained trees) remains on the site, and the net-production area is reduced in the long term. At the level of forest landscapes with multiple owners and different types of ownership, an increased production by some of the forest owners could compensate for any reduction in production incurred by one or several other owners. Current average harvesting rates in temperate Europe are commonly below the sustained yield, which in combination with an expansion in forest area, N deposition, improved management and other environmental drivers have led to an increase in growing stocks in the region (Forest Europe 2015). This indicates that the trade-off between wood production and retention may be partially resolved by applying tailored management strategies across forest landscapes, e.g. intensifying wood production in areas with high productive potential, while retaining ecologically important structural elements where appropriate, and avoiding possible leakage effects (e.g. substitution of wood by other materials or increasing imports). The impacts of such a patchwork of small-scale retention and intensification on biodiversity conservation could be positive compared to the status quo (Schall et al. 2018).

Studies on the implementation of retention forestry by public forest managers (Maier and Winkel 2017) or, broader, of Natura 2000 (including retention approaches) have focused on socio-economic and political conditions that greatly determine the effectiveness “on the ground” (Winter et al. 2014). Economic perspectives for assessing the cost of retention practices in CCF have also been proposed. These analyses typically assess the opportunity costs (foregone revenue because of not harvesting timber) related to habitat trees and deadwood (e.g. Rosenkranz et al. 2014; Augustynczik et al. 2018) but have also evaluated the increased administration and transaction costs related to tree marking, monitoring and work safety (Smitt et al. 2017). Nevertheless, there is still a lack of studies aiming at identifying optimal retention levels. This kind of research necessitates a deeper analysis of both the preferences and values of all stakeholders, as well as the dynamics of the environmental system under study.

## Monitoring

No large-scale monitoring of the effectiveness of ecological structures resulting from retention has yet been implemented involving the whole of Central Europe or even several countries. It is difficult to make comparisons on retention levels between countries, forest management systems or forest types using European certification standards (Appendices S2, S3) since quantitative targets regarding the number of habitat trees and amounts of deadwood are not typically specified. Nevertheless, some monitoring initiatives exist, and range from the individual landowner to state level. Habitat trees are marked and followed in both private forests and public land (Fig. 2). One regional example covering different forest owners is the German State of Baden-Württemberg where the last national forest inventory has registered habitat trees defined by the presence of certain microhabitats (Appendix S4). National forest inventories are mostly based on in situ grid-point sampling and might prove insufficient since rare retention elements may not be adequately captured within the sampling units (e.g. Bäuerle et al. 2009). However, advancements in remote sensing techniques, including terrestrial LiDAR systems (Seidel et al. 2016), and SfM (structure from motion; Frey et al. 2018), might present future possibilities for better monitoring of retention elements (Hirschmugl et al. 2007).

## Ways forward

### Improving socio-economic incentives

The implementation of retention forestry is critically dependent on both the broader socio-economic and political setting and individual capabilities of forest managers and forest owners. Economic incentives may be required to resolve trade-offs between wood production and retention (Augustynczik et al. 2018), from different types of payment for environmental services, particularly on private lands, to the adjustment of timber production targets and economic goals for public lands (Maier and Winkel 2017). In this sense, voluntary compensation schemes to support retention forestry could supplement public policies, increasing the efficiency and reducing public expenditure on conservation programs (Chobotova 2013). Payments supporting biodiversity conservation need to consider not only forgone timber value, but also changes to other aspects of management, including inventory, planning, and work safety. Moreover, “soft” instruments that provide digestible information on the importance of retention, effectively spread this knowledge through different channels among forest managers, or offer the possibility of consulting biodiversity experts within the forest services will be essential elements of implementation (Bieling 2004; Maier and Winkel 2017). In so doing, foresters who have been traditionally trained to eliminate trees that are less valuable from an economic standpoint might instead be guided to view the trees as having high microhabitat potential.

The most significant advancement, in fact, would be the development of a forestry culture in which managers move from maintaining habitat trees either out of necessity or compliance with legal requirements to implementing retention practices with a sense of purpose. This shift in culture must be accompanied by beneficial changes in socio-economic and policy incentives at higher levels of decision-making, and by a greater appreciation of the value of biodiversity. Otherwise, unsupported incentives can only foster a defensive attitude towards environmental demands that inevitably will prevent forest managers from feeling empowered to implement retention (Sotirov and Winkel 2016).

### Spreading risks in the era of climate change

Retention strategies, as with other forest management operations, need to be adapted to the uncertainty associated with climate change. It is well understood that structural diversity may be a way to adapt to climate change since structurally diverse forests may be more resistant to disturbance (Pretzsch et al. 2018, but see Dănescu et al. 2018). This could be partly achieved by encouraging the natural regeneration of retention trees belonging to tree species that might be better adapted to future environmental conditions. Furthermore, diversity in the retained tree species should also be considered, as this could decrease the risk of species collapse from potential pest and pathogen disturbances, e.g. European ash dieback (Gross et al. 2014). Currently rare tree species that are well adapted to warmer and drier conditions in Europe such as those from *Sorbus* and *Acer* genera could be another viable option for habitat trees since they directly benefit biodiversity and may increase the overall resilience of the forests to global changes (Bauhus et al. 2017).

### Adapting to ownership complexity

Both the spatial arrangement of retention elements and their functional connectivity at the landscape level are shaped by variation in forest property sizes and management approaches. Although retention approaches are more likely to be implemented on public land, monitoring data show (Appendix S4) that private forests often have high biodiversity qualities (see also Johann and Schaich 2016). Here, incentives are required so that private forest owners maintain these conservation values. In addition, new management models are needed that integrate the goals of these two owners, to allow large-scale planning that includes different types of production forests and sets aside from single retained trees to large reserves. One way to develop such novel governance models would be to design study landscapes in different regions and then build on the gained experience.

### Importance of disturbances

There is a need for retention practices in CCF to place a larger emphasis on trees in gaps, edge zones and other open spaces. Apart from large areas with late-successional stages, disturbed and more open forest belong to the natural landscape of temperate forests in Europe (Nagel et al. 2014). Consequently, habitat is needed for species in old, closed forests but also for species adapted to sunny and open conditions (Seibold et al. 2015). In CCF, where a major goal of management is to decrease disturbances such as windthrow, the need for early successional habitats is sometimes forgotten. The importance of disturbances in relation to retention strategies has been increasingly recognized (Gustafsson et al. 2012) although the major original aim was the maintenance of old-forest conditions across logging cycles, namely the life-boating function (Franklin et al. 2000).

### Development of on-site retention strategies

The selection of habitat trees normally occurs at the onset of the harvesting phase (Fig. 2). However, many potential habitat trees, e.g. uncommon tree species or unusual tree forms and their associated microhabitats, will have already been removed during thinning. Thus, it is important that foresters apply tending regimes during early stand development that include the selection of future habitat trees, for example dominant trees with special forms, forked or with strong branches from which hollows may develop (Bauhus et al. 2009). The latest forest inventory of Baden-Württemberg, Germany shows that most designated habitat trees are in early-development stages; only 4% of the mapped habitat trees support more than two attributes like hollows, bracket fungi, large bird nests, stem rot and loose bark or bark pockets (Appendix S4). In the selection of habitat trees, it will be important to prioritize trees with stable root systems positioned in a sheltered surrounding, to avoid the risk of windthrow. Further development of criteria for the identification and selection of habitat trees is needed.

A broad desirable future development is a move away from the strong focus on retaining habitat trees, to also increasingly retaining larger patches of forests, which, according to species-area predictions (Gleason 1922), may support greater biodiversity. Sites with particular topography, soils and hydrology are then especially valuable. Ideally, advice should be given to forest owners on thresholds for structures important to biodiversity, including minimum number old trees per ha. Unfortunately, the scientific knowledge to support such targets is still too weak to allow for generalizations. Clearly, the retention targets applied today, as shown in certification standards (Appendix S2), are far below the quantity and quality of habitats found in natural forests or long-term forest reserves (e.g. Paillet et al. 2017). Thus, from a biodiversity conservation point of view, until we have stronger evidence-based information the general rule is “the more the better”. Nevertheless, we fully recognize the need for policy makers and managers to set up and work towards targets. One solution would be to seek advice from expert panels of forest ecologists and conservation biologists, who can, in the absence of strong scientific evidence, provide information based on practical experience and observations and suggest ways to deal with the uncertainty about clear quantitative targets.

Ideally, future retention strategies, like relative prioritization of habitat trees versus dead wood or individual trees versus tree patches, should be adapted to the variation in forest types and their states in temperate forests of Europe. CCF is being practised with a variety of silvicultural systems throughout the region (Brang et al. 2014), and in production forests comprising tree species such as *F. sylvatica*, *P. abies*, *P. sylvestris*, *Abies alba*, and also species belonging to *Quercus*, *Fraxinus* and *Acer*. Yet, the approaches to retention forestry, where it has been implemented, are quite similar. Future forest monitoring and research may give more detailed guidance on the need to maintain and restore key processes and structural components of importance to biodiversity, and how these vary among forest types, silvicultural systems and regions.

## Conclusions

Our overview shows that the application of retention approaches to CCF in temperate forests of Europe offers a promising conservation approach complementary to strict forest reserves. There is an evident potential to expand retention measures in production forests, which dominate the European forest land base. Favourable and supportive socio-economic and governance frameworks are needed to include more forestland as well as to increase the density of retention structures. Increased rates of implementation will depend on the combination of effective legislation and economic incentives (to at least compensate for opportunity costs of retention), as well as the development of a culture that fosters interest and learning related to retention. Understanding the fundamental role biodiversity plays in maintaining resilient forests is imperative for motivating practitioners. Although previous research has provided general strong support for the ecological relevance of the retention forestry model, there is a definite need for more specific studies on the role of retention in CCF. A review of studies on biodiversity associated with habitat trees and deadwood, also in relation to variations in light availability in CCF stands, would give guidance on more specific prioritization and management of such elements. In the end, the design of efficient CCF retention strategies hinges on the combination of ecological and socio-economic knowledge.

## Electronic supplementary material

Below is the link to the electronic supplementary material.

Supplementary material 1 (PDF 397 kb)
