# Supplementary material for: Retention as an integrated biodiversity conservation approach for continuous-cover forestry in Europe
Source: Ambio. 2019 May 4;49(1):85–97. doi: 10.1007/s13280-019-01190-1 (PMC6889099; doi:10.1007/s13280-019-01190-1)
Supplement: Supplementary file 1 — Supplementary material 1 (PDF 397 kb) [file 13280_2019_1190_MOESM1_ESM.pdf]

Ambio

Electronic Supplementary Material
This supplementary material has not been peer reviewed

Title: **Retention as an integrated biodiversity conservation approach for continuous-cover forestry in Europe**

Lena Gustafsson, Jürgen Bauhus, Thomas Asbeck, Andrey Lessa Derci Augustynczik, Marco Basile, Julian Frey, Fabian Gutzat, Marc Hanewinkel, Jan Helbach, Marlotte Jonker, Anna Knuff, Christian Messier, Johannes Penner, Patrick Pyttel, Albert Reif, Felix Storch, Nathalie Winiger, Georg Winkel, Rasoul Yousefpour, Ilse Storch

Appendix S1. Terms related to retention approaches in forestry

Clearcutting with reserves	"A variation of clearcutting in which trees are retained either uniformly or in small groups, for purposes other than for regeneration" (British Columbia Ministry of Forests 2003).<br><br>Helms (1998) defines reserve tree as "a tree, pole-sized or larger, retained in either a dispersed or aggregated manner after the regeneration period under the clearcutting, seed tree, shelterwood, group selection or coppice methods." Historically, reserves were used mostly with the seed tree system when selected stems were left with the intention of obtaining further increment from them before eventual harvesting (Smith 1962, Matthews 1989).
Green-tree retention	Green trees and retention are mentioned in several publications by Jerry Franklin and colleagues from the mid-late 1980s (e.g., Franklin et al, 1986). A thorough description of green-tree retention is presented in Franklin (1992). The term has been largely replaced in recent decades with variable retention.
Retention Forestry	This concept was introduced at a workshop in Sweden with researchers and practitioners in 2011 (Gustafsson et al. 2012), but was used occasionally before that, e.g., in Franklin et al. (2000). The term is mostly used in the scientific literature and less in practical forestry.
Retention System	A silvicultural system introduced in British Columbia regulations in 1999 as the primary system for implementing variable retention. The definition and rationale for the retention system is described by Mitchell and Beese (2002). The retention system is designed to "retain individual trees or groups of trees to maintain structural diversity over the area of the cutblock for at least one rotation, and leave more than half the total area of the cutblock within one tree-height from the base of a tree or group of trees" (i.e., maintain >50% forest influence) (British Columbia Ministry of Forests 2003).
Structure/structural retention	Structure retention is used within practical forestry in parts of Canada, like Alberta (Alberta Forest Management Planning Standard 2006). Structural retention is sometimes used in the research literature, e.g. Franklin et al. (2002),
Variable retention	The Scientific Panel for Sustainable Forest Practices in Clayoquot Sound, British Columbia, Canada (CSSP 1995) suggested this term in the mid-1990s in their strategy for harvesting temperate rainforests, after which this approach was largely adopted (Mitchell & Beese 2002, see also Franklin et al. (1997). This term is broadly used in practice and research in many parts of Canada, the US, and Australia, but less so in Europe.
New Forestry	A broad, holistic term for a forestry model introduced in the late 1980s in the Pacific NW of North America which emphasizes considering patterns and processes of natural disturbance dynamics with the retention of dead and living trees as a key tenet (Franklin 1989). Related terms are sustainable forestry and ecosystem management (Franklin 1998). Not much used today.

Literature cited

Alberta Sustainable Resource Development. 2006. Alberta forest management planning standard, version 4,1, Alberta Sustainable Resource Development, Alberta, Canada. Available at: http://www1.agric.gov.ab.ca/$department/deptdocs.nsf/all/formain15749/$FILE/ForestManagementPlanningStandard-2006.pdf.

Bohn U, Neuhäusl R, Hettwer C, Neuhäuslová Z, Schlüter H, Weber HE. 2003. Karte der natürlichen Vegetation Europas / Map of the Natural Vegetation of Europe, Maßstab / Scale 1 : 2500000. Landwirtschaftsverlag, Münster, Germany.

British Columbia Ministry of Forests. 2003. Silvicultural systems handbook for British Columbia. British Columbia Ministry of Forests. Victoria, BC, Canada, Available at: http://www.for.gov.bc.ca/HFP/Pubssilvsystems.htm

Clayoquot Sound Scientific Panel. 1995. Sustainable ecosystem management in Clayoquot Sound: planning and practices. Cortex Consultants Inc., Victoria, BC, Canada, Available at: http://blogs.ubc.ca/apfnet01/files/2015/08/Clayoquot_Science_Panel_Report-5-Sustainable_Ecosystem_Management.pdf

Franklin JF, Spies TA, Perry D, Harmon M, and McKee A. 1986. Modifying Douglas-fir management regimes for nontimber objectives. Douglas-fir: stand management for the future. Proceedings of Symposium. June 18-20, 1985. University of Washington, Seattle, WA. Contrib, No. 55, College of Forest Resources, University of Washington, Seattle, WA. Pp, 373-379.

Franklin JF. 1989. Towards a new forestry. American Forests **95**:37–44.

Franklin JF. 1998. The natural, the clearcut, and the future. Northwest Science **72**:134-138.

Franklin JF. 1992. Scientific basis for new perspectives in forests and streams. Pages 25-72 in Naiman RJ. editor. Watershed management: balancing sustainability and environmental change. Springer-Verlag, New York, NY.

Franklin JF. Berg DR. Thornburgh DA. Tappeiner JC. 1997. Alternative silvicultural approaches to timber harvesting: variable retention harvest systems. Pages 111-140 in Kohm KA & Franklin JF, editors. Creating a forestry for the 21st century. Island Press, Washington, D.C., USA.

Franklin JF. et al. 2000. Threads of continuity. Conservation **1**:8-17.

Franklin JF. et al. 2002. Disturbances and structural development of natural forest ecosystems with silvicultural implications, using Douglas-fir forests as an example. Forest Ecology and Management **155**:399-423.

Gustafsson L. et al. 2012. Retention forestry to maintain multifunctional forests: a world perspective. BioScience **62**:633–645.

Hansen MC. et al. 2013. High-resolution global maps of 21st-century forest cover change. Science **342**:850-853.

Helms JA (Ed.). 1998. Dictionary of forestry. Society of American Foresters. Bethesda, MD, USA.

Matthews JD. 1989. Silvicultural systems. Oxford University Press. Oxford, UK.

Mitchell SJ & Beese WJ. 2002. The retention system: reconciling variable retention with the principles of silvicultural systems. The Forestry Chronicle **78**:397-403.

Smith DM. 1962. The practice of silviculture. 7th ed. John Wiley & Sons. New York, NY, USA.

Appendix S2. The retention prescriptions (per hectare) mandated by certification standards (PEFC/FSC) in European countries. Source: Endorsed national PEFC/FSC standards as published on https://pefc.org/standards/national-standards/endorsed-national-standards (Accessed 13 Dec 2017), https://ic.fsc.org/en/document-center (Accessed 13 Dec 2017). Information is lacking on the forest management systems in respective country, and thus retention prescriptions specific to continuous-cover forestry cannot be assessed. For information on the certified forest areas of specific countries, see Appendix S3

Country	PEFC Living habitat trees	FSC Living habitat trees	PEFC Standing and downed deadwood amount – trees	FSC Standing and downed deadwood amount – trees
Austria	+		+	
Belarus	5			
Belgium	1		1	
Bulgaria		+		+
Czech Republic	-	5	+	5
Denmark	5	3	3	3
Estonia	+		+	
Finland	10	10	10	20
France	1	2	1	+
Germany	+	10	+	+
Hungary	3		+	
Ireland	+	+	+	**
Italy	-	-	-	+
Kosovo		+		+
Latvia	+		+	
Luxembourg	+	+	+	+
Netherlands	+	+	8	4
Norway	10		+	
Poland	+	+	+	***
Portugal	+	+	+	+
Russia		+		+
Slovakia	5		+	
Slovenia	-		+	
Spain	-		+	
Sweden	10	10	+	+
Switzerland	5		**	
UK	-	+	-	**

- : No information, +: qualitative retention amounts (“some”, “adequate number”), empty cells: No endorsed national standard provided at time of data assessment

** Switzerland PEFC deadwood: 15m³/ha, Ireland FSC deadwood: 8m³/ha, UK FSC deadwood: 20m³/ha

*** Poland FSC deadwood: minimum area 0.05 ha per ha

Appendix S3. Additional information on certified forest areas in European countries

The PEFC and FSC certified forest areas, as well as total forest area (in ha) in various European countries. Data regarding PEFC and FSC certified areas are from 2017, while data on total forest area are from 2015. In some cases, the total forest area falls below the total certified forest area (PEFC and FSC) due to differences in the definition of a forest between sources. In some cases, forest properties are double-certified, i.e. according to PEFC as well as FSC, which means that forest area figures cannot be summed.

	PEFC certified area (ha)[1]	FSC certified area (ha)[2]	Total forest area (ha)[3]
Austria	3,111,137	587	3,869,000
Belarus	8,710,234	8,281,505	8,633,500
Belgium	299,357	23,621	683,400
Bulgaria	NA	1,325,808	3,823,000
Czech Republic	1,811,407	52,729	2,667,000
Denmark	264,18	212,739	612,200
Estonia	1,217,631	1,428,797	2,232,000
Finland	17,784,457	1,478,032	22,218,000
France	8,096,117	43,423	16,989,000
Germany	7,424,185	1,156,053	11,419,000
Hungary	NA	302,286	2,069,000
Ireland	376,108	446,222	754,020
Italy	745,186	63,744	9,297,000
Kosovo	NA	NA	NA
Latvia	1,700,889	1,022,196	3,356,000
Luxembourg	34,677	22,062	86,700
Netherlands	3.240	178,086	376,000
Norway	7,380,750	444,654	12,112,000
Poland	7,252,197	6,936,266	9,435,000
Portugal	253,657	384,588	3,182,000
Russia	13,180,950	41,913,942	814,930,500

Slovakia	1,229,324	146,832	1,940,000
Slovenia	283,319	264,971	1,248,000
Spain	2,153,431	260,671	18,417,870
Sweden	11,549,700	12,255,794	28,073,000
Switzerland	208,949	616,13	1,254,000
UK	1,409,761	1,619,519	3,144,000

[1] https://www,pefc,org/images/documents/PEFC_Global_Certificates_-_Dec_2017,pdf - accessed 28,3,2018

[2] https://ic,fsc,org/en/facts-and-figures - accessed 28,3,2018

[3] http://www,fao,org/faostat/en/#data/GF - accessed 28,3,2018

Appendix S4. The size distribution of habitat trees assessed in the third national forest inventory of Baden-Württemberg, Germany.

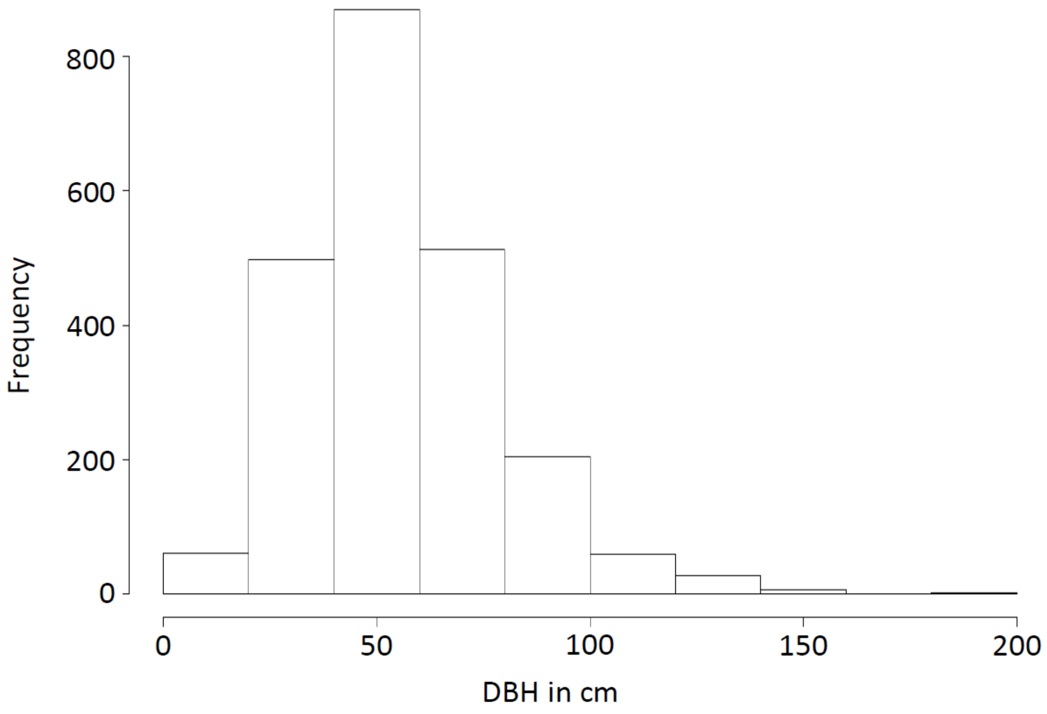

The most recent Forest Inventory (BWI 3, 2012) assessed trees with special attributes, among which were a number of microhabitats that permit the identification of trees as habitat trees. These microhabitats included tree hollows, presence of bracket fungi, presence of large bird nests (> 50 cm diameter), stem rot (> 500 cm^2 surface), loose bark or bark pockets (> 500 cm^2 surface), a large stem mold cavity, heavy sap or resin flow, significant crown dead wood (> 1/3 of upper crown or > 3 branches > 20 cm diameter). In addition to trees with these attributes, marked habitat trees were

also recorded. In the state of Baden-Württemberg, a total of 99,950 trees were assessed, out of which 2,236 showed one or more of the above special attributes. This translates into an average frequency of 5.2 habitat trees (HT) per ha, which is just above the target of five habitat trees per ha recently proposed in the Old and Dead Wood Concept developed for state forests (Forst BW 2016). Since the aforementioned attributes are not as comprehensive as other recently developed lists of microhabitats that can be used to characterize habitat trees (e.g. Larrieu et al. 2018), these figures may be regarded as a conservative estimate. These average figures also illustrate that the challenge for forest management is not only to generate habitat trees, but also to retain them. However, since only 4 % of all habitat trees supported more than two of the special features, the majority of the classified individuals may be regarded as “early mature habitat trees”. It can be expected that many of them will develop more microhabitat features with time. In addition, habitat trees were not evenly distributed. Whereas 9.1 HT ha^{-1} were noted for broadleaved forests, there was an average of only 2.8 HT ha^{-1} in coniferous forests. Remarkably, the average HT density per ha in private forests (5,0) was slightly higher than that in state forests (4.6); in municipal forests, the average was 5.5. These figures highlight the important role that private forests can play in landscape-level biodiversity conservation. In the youngest stand development phase (avg DBH < 20 cm), the HT density was substantially lower (1.8 HT ha-1) than in all other stand development phases (DBH 20-35 cm: 5.0 HT ha^{-1}; DBH 35-50 cm: 6.2 HT ha^{-1}; DBH 50-70 cm: 6.9 HT ha^{-1}; DBH >70 cm: 10.9 HT ha^{-1}). These figures indicate a lack of habitat trees in young forests and that no effective retention forestry practices have been implemented in the past.

Literature cited

ForstBW. 2016. Alt- und Totholzkonzept Baden-Württemberg, Landesbetrieb ForstBW. Stuttgart, Germany. Available at: http://www.forstbw.de/fileadmin/forstbw_infothek/forstbw_praxis/ForstBW-PRAXIS_Alt-_und_Totholz_WEB.pdf

Larrieu L. et al. 2018. Tree related microhabitats in temperate and Mediterranean European forests: a hierarchical typology for inventory standardization. Ecological Indicators **84**:194-207.

Appendix S5. High-resolution maps of the European distribution of temperate forests.

Maps can be downloaded from https://drive.google.com/drive/folders/1rctI84iSamKZ-dSyTKhChagyVB8dZUPp?usp=sharing. Maps show, in detail, the potential natural distribution of temperate forests (dark grey) in Europe (after Bohn et al. 2000/2003). This is overlaid with the actual forest cover (in four classes, colored from light to dark green: >10-40%, >40-60%, >60-80%, >80-100%). Forest cover information was derived from satellite imagery (MODIS data, from Hansen et al. 2013). Land masses are depicted in light grey.

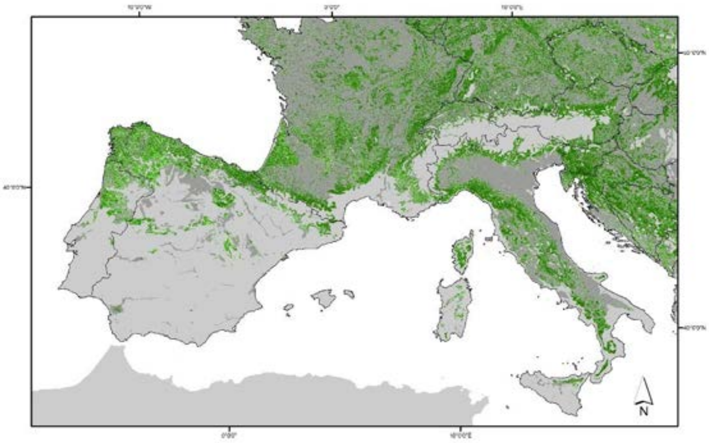
N

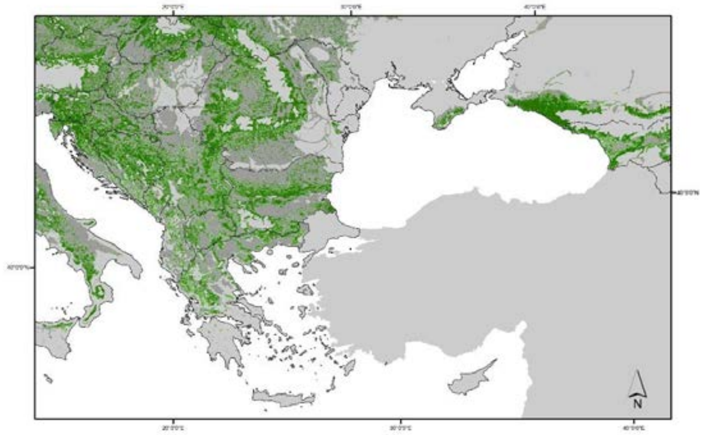
N

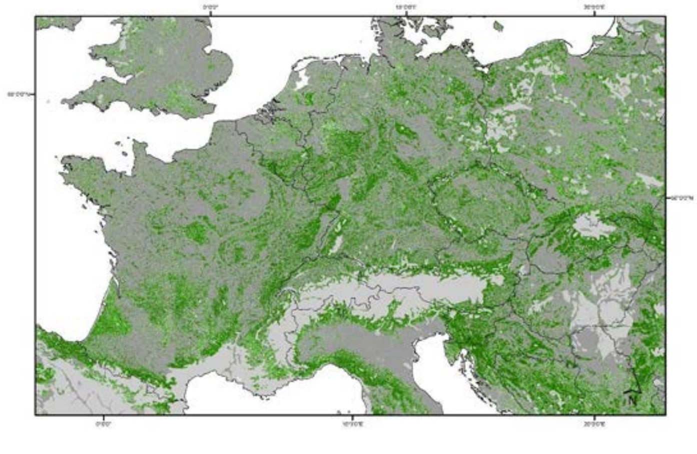
N

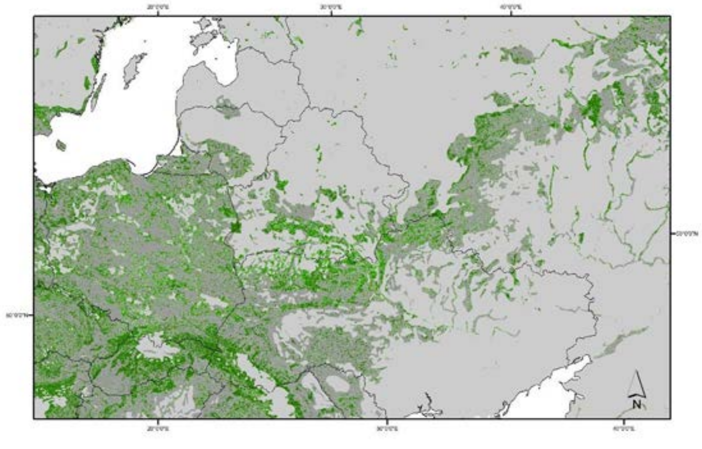
N

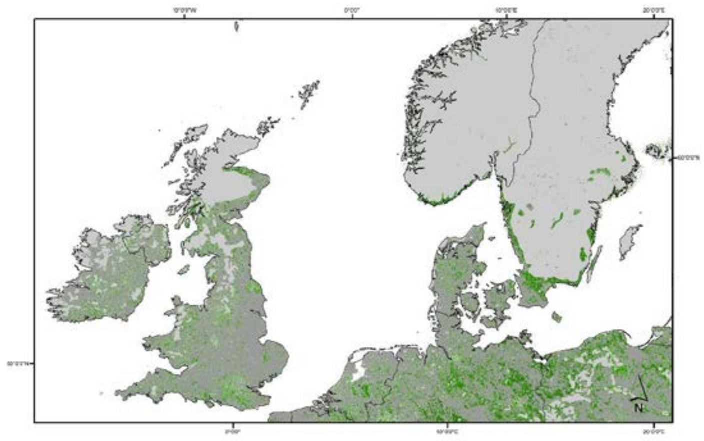

Appendix S6. Additional information on examples of retention approaches used in continuous cover forestry in different parts of Europe

DENMARK. 18% (110,000 ha) of Denmark's forests belong to the state (managed by The Nature Agency) and, during the last century, have mainly been managed as even-aged forests with Norway spruce (*Picea abies*), beech (*Fagus sylvatica*) and oak (*Quercus robur*) as the dominating species. According to an action plan launched in 2005, the forests are to be converted into uneven-aged, deciduous-dominated stands through close-to-nature management, with a transition time of one to two tree generations. This implies a change to single tree and group selection with the use of natural regeneration. Uncommon, indigenous tree species will be promoted and the use of pesticides and fertilizers is now prohibited. A 2016 decision states that 20% of state forests are to be set aside for conservation and restored to more natural conditions during a 10-year period. For the remaining 80%, which are managed as production forests, Danish Nature Agency guidelines state that retention actions should be taken at harvesting. The main actions include leaving at least five living trees per ha, as well as hollow trees, deciduous trees with cavities, and other valuable nesting trees, the retention of all oaks >300 years old and beeches >200 years old, intentional damage to three trees per ha 20-40 cm in diameter at breast height in middle-aged deciduous stands (see photo in Figure 2), leaving groups of old trees standing as long as possible, and promoting non-commercial tree species and shrubs in the understory.

SW GERMANY: A private forest owner in the Black Forest region has applied retention measures to his 230 ha of land for the last 15 years. Habitat trees, especially those with cavities, are marked (see photo in Figure 2) and excluded during harvesting, which follows a five-year interval, with the marked trees still visible at the time of the following harvesting operation. A one ha patch with old trees and high amounts of deadwood has been set aside in a 155-year-old beech stand, with the main aim being conservation of the bat species *Plecotus auritus*. An energy company reimburses the forest owner, as the patch lies within a compensation area for a small-scale wind park in the vicinity.

NE GERMANY. In the German federal state of Brandenburg, the Methusalem project has been implemented in 436,000 ha of public forest. The project aims to increase the number of old and beautiful trees for both nature conservation and aesthetic reasons. Up until today, more than 200,000 trees have been selected, with a minimum of five trees per ha. About 70% of all selected trees are Scots pines, followed by oaks and other broadleaf species. Living trees of low economic value are preferably selected. All trees are permanently marked, numbered, and described by the responsible forest authorities. Certain patches > 1000 m² are also left for natural development.

ITALY Elements of retention forestry were introduced in the Molise Region, central Italy, about 10 years ago. The approach covers Natura 2000 sites, managed through continuous-cover forestry, comprising 3,100 ha. Initial regulations included retention of at least two living and two dead trees per ha, as well as all living or dead cavity trees. Following evaluation, retention prescriptions have subsequently been developed to include at least two trees per ha in downy oak stands, three trees per ha in sessile oak stands and five trees per ha in any other stand, as well as saving all standing dead trees. One specific aim of retaining old living trees is to provide habitat for species listed in the EU Habitat directive, i.e. great capricorn beetle *Cerambyx cerdo*. The photo in Figure 2 shows the lower part of a beech tree *Fagus sylvatica* close to a stream. Cavities between the buttresses and the pockets between the ivy and the stem form important microhabitats for the spectacled salamander *Salamandrina perspicillata*, also listed in the EU Habitat directive.
